# A statistical frame based TDMA protocol for human body communication

**DOI:** 10.1186/s12938-015-0061-1

**Published:** 2015-07-09

**Authors:** Zedong Nie, Zhao Li, Renwei Huang, Yuhang Liu, Jingzhen Li, Lei Wang

**Affiliations:** Shenzhen Institutes of Advanced Technology, Chinese Academy of Sciences, Shenzhen, 518005 China

**Keywords:** Human body communication, Wireless body area networks, Media access control, Time division multi access, Statistical frame

## Abstract

**Background:**

Human body communication (HBC) using the human body as the transmission medium, which has been regarded as one of the most promising short-range communications in wireless body area networks (WBAN). Compared to the traditional wireless networks, two challenges are existed in HBC based WBAN. (1) Its sensor nodes should be energy saving since it is inconvenient to replace or recharge the battery on these sensor nodes; (2) the coordinator should be able to react dynamically and rapidly to the burst traffic triggered by sensing events. Those burst traffic conditions include vital physical signal (electrocardiogram, electroencephalogram etc.) monitoring, human motion detection (fall detection, activity monitoring, gesture recognition, motion sensing etc.) and so on. To cope with aforementioned challenges, a statistical frame based TDMA (S-TDMA) protocol with multi-constrained (energy, delay, transmission efficiency and emergency management) service is proposed in this paper.

**Methods:**

The scenarios where burst traffic is often triggered rapidly with low power consumption and low delay is handled in our proposed S-TDMA. A beacon frame with the contained synchronous and poll information is designed to reduce the possibility of collisions of request frames. A statistical frame which broadcasts the unified scheduling information is adopted to avoid packet collisions, idle listening and overhearing. Dynamic time slot allocation mechanism is presented to manage the burst traffic and reduce the active period in each beacon period. An emergency mechanism is proposed for vital signals to be transmitted. The theory analysis is proceed and the result is evaluated in the hardware platform.

**Results:**

To verify its feasibility, S-TDMA was fully implemented on our independently-developed HBC platform where four sensor nodes and a coordinator are fastened on a human body. Experiment results show that S-TDMA costs 89.397 mJ every 20 s when the payload size is 122 bytes, 9.51% lower than Lightweight MAC (LMAC); the average data latency of S-TDMA is 6.3 ms, 7.02% lower than Preamble-based TDMA (PB-TDMA); the transmission efficiency of S-TDMA is 93.67%, 4.83% higher than IEEE 802.15.6 carrier sense multiple access/collision avoidance (CSMA/CA) protocol.

**Conclusions:**

With respect to the challenges of HBC based WBANs, a novel S-TDMA protocol was proposed in this paper. Compared to the traditional protocols, the results demonstrate that S-TDMA successfully meets the delay and transmission efficiency requirements of HBC while keeping a low energy consumption. We also believe that our S-TDMA protocol will promote development of HBC in wearable applications.

## Background

With the rapid development of sensors and wireless communication technologies, the wireless body area network (WBAN) provides a promising solution for mobile health [1–3]. Meanwhile, IEEE 802 established a Task Group called IEEE 802.15.6 for the standardization of WBAN [4]. Recognized by IEEE 802.15.6 standard for WBAN as a physical (PHY) layer, human body communication (HBC), which uses the human body as a propagation medium, is a promising communication solution for WBAN. HBC has advantage of lower power consumption, higher data rate and security [5, 6]. HBC based WBAN is made up of a collection of sensor nodes and a resource-rich data aggregation device as a coordinator. The sensor nodes and the coordinator are fastened on a human body [7, 8]. Wearable or implanted sensors nodes can sample, process and transmit signals and exchange signals with the coordinator through HBC. Compared to the traditional wireless technologies based WBAN, four special concerns should be considered in WBAN which HBC is adopted: (1) their sensor nodes are fixed on/in-body, so they are free of mobility; (2) distance between two sensor nodes are always within the arm span of a person (<2 m), making them less power consuming to synchronization [9, 10]; (3) sensor nodes must be equipped with batteries in small dimension that offer continuous power for a couple of months or even several years [11, 12]. (4) The burst sensing events like emergency alerts, critical physiological data, and multimedia streams, are often happened and trigger traffics [13, 14]. Considering the aforementioned factors, how to minimize the power consumption of the sensor nodes and how to manage the burst data services effectively become two important issues, which are controlled and handled by MAC protocol [15]. In other words, changes in the load of the sensor nodes should be handled dynamically and rapidly, requiring low data latency and high transmission efficiency of MAC protocols [16–21]. Therefore, it is required to design a MAC protocol to efficiently solve the power consumption and emergency traffic problems in HBC based WBAN.

So far, a number of potential contributions to WBAN designs can be found in [22–37], which focus on energy-efficient and low-latency MAC solutions. One solution is based on contention. Carrier sense multiple access (CSMA) is a classical contention protocol in which a node verifies the absence of other traffic before transmitting [22, 23]. However, the packet collisions, overhearing and idle listening that exist in CSMA protocols make CSMA quite energy consuming [24]. In order to avoid collisions, idle listening and overhearing, carrier sense multiple access/collision avoidance (CSMA/CA) protocol is introduced in IEEE 802.15.6 and IEEE 802.15.4. The performance of a non-beacon IEEE 802.15.4 is studied in [25], where low upload/download rates are considered. However, their work only considers scenarios where sensors nodes report data periodically to the coordinator, making it not flexible to be applied to high data rate and real-time applications. In [26], performances of IEEE 802.15.4 is studied when the power management mechanism is enabled, it was concluded that 802.15.4 MAC is not appropriate for applications with very stringent latency requirements, as it is not able to guarantee an acceptable reliability level subject to the required timeliness. As to IEEE 802.15.6 CSMA/CA, having short exclusive access phases (EAP) and random access phases (RAP) lead to its inefficient use of bandwidth, especially result in high traffic loads [27]. In addition, CSMA/CA based protocols have significant overhead that decreases transmission efficiency [28, 29]. Other protocols based on contention such as Collaborative MAC (CC-MAC) [30] and STEM protocol [31] are not practical since their complexity and requirement for extra hardware. Moreover, contention-based solutions are not applicable to the situation where traffic is correlated. For example, a patient suffering from heart disease triggers pulse signal, blood pressure signal and blood oxygen signal [32]. These kinds of physiological parameters increase the traffic correlation. On such circumstances, a contention-based protocol will incur heavy collisions leading to extra energy consumption. Another solution is based on time division multi access (TDMA). Many researchers have developed quite a few TDMA based protocols with dynamic time slot allocation, such as preamble-based TDMA (PB-TDMA) protocol [33] and lightweight MAC (LMAC) protocol [34]. PB-TDMA protocol outperforms other protocols since it does not need a coordinator to manage the whole network. However, during preamble period each node has to bring the information of synchronization and request which contribute to energy expenditure. LMAC utilizes one time slot to assign control message and data to each node in order to control it during this time slot. Nevertheless, these two protocols are not energy-efficient since there are too many control messages in their frames which results in large overhead and high data latency [35]. In [36], a TDMA based protocol is proposed which assumes that the topology of WBAN is fixed. This assumption makes the protocol unable to adapt to the associations and dissociations of sensor nodes in the network. In [37], a superframe structure, which allows diverse periods for diverse traffic classes according to their respective quality of service (QoS) requirements. However, the emergency mechanism in the protocol can lead to a delay of a superframe period which is not suitable to be used in burst traffic. To sum up, although most of the works on MAC protocol for WBANs focused on energy-efficiency, latency, transmission efficiency and emergency, few MAC protocols could take into account all the issues above together.

Considering the special factors that exist in HBC based WBAN, a statistical frame based TDMA protocol for HBC is proposed in this paper. First of all, a beacon frame with the synchronous and poll information is constructed for reducing the possibility of collisions of request frames. Then, a statistical frame is designed to broadcast the unified scheduling information, which would contribute to avoiding packet collisions, idle listening and overhearing. Next, a novel dynamic time slot allocation mechanism is developed to ensure packet delivery with the least possible delay and consumption. Finally, a superframe structure for statistical frame based time division multi access (S-TDMA) is created, and the analytical calculation and extensive experiments are performed.

The contributions of this paper could be summarized as follows:

- To avoid conflicts, idle listening and overhearing in data transmitting, a novel superframe structure with a statistical frame is designed;
- To reduce the active period in each beacon period and to meet the requirement of the application targeted by the IEEE 802.15.6 standard, dynamic time slot allocation is proposed to handle the irregularly changes of the load in each sensor node;
- To evaluate the performance of our proposed protocol, the theory analysis is proceed and the protocol is implemented in a real WBAN platform. Results show that in comparison to PB-TDMA protocol, LMAC protocol and IEEE 802.15.6 CSMA/CA protocol, S-TDMA can be energy saving with high transmission efficiency and low data latency.

## Methods

### Protocol design

Most WBAN presented in the works have a single-hop star topology with a personal digital assistant (PDA) or a personal computer (PC) serving as the network coordinator [38, 39]. Therefore, a single-hop star network based on HBC technique is implemented on our WBAN development platform. In order to make S-TDMA energy-efficient, both TDMA-based scheme and unified scheduling are applied to it. In this Section, we describe the frame format and allocation scheme of S-TDMA protocol.

#### Superframe structure

**Figure 1:**
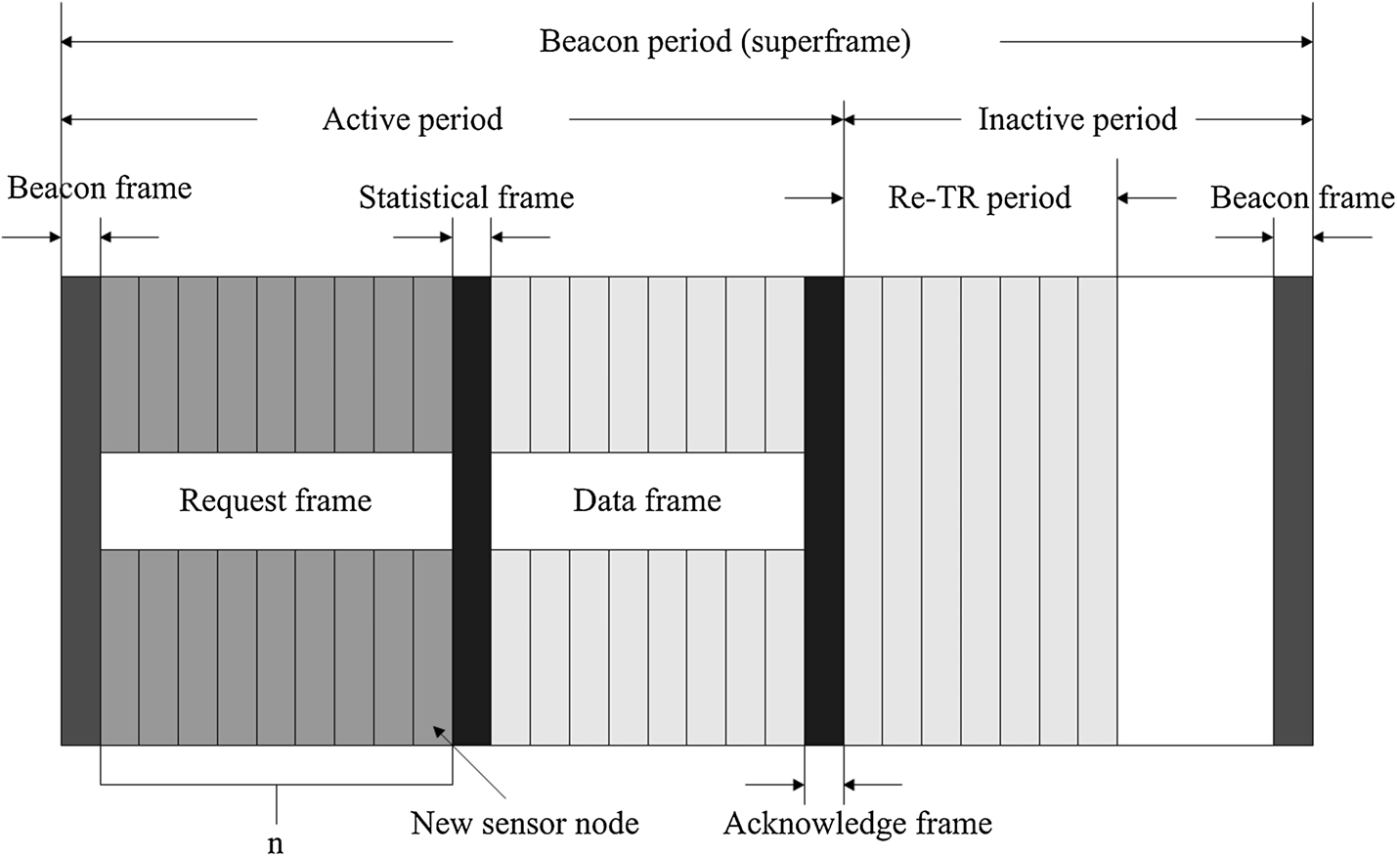
Structure of the superframe used in S-TDMA. Active period and inactive period are included in this superframe. Active period is made up of beacon frame, request frame, statistical frame, data frame, and acknowledge frame; while inactive period consists of retransmission frame.

The beacon period of the MAC superframe lasts for 1 s and consists of two periods: active period and inactive period as illustrated in Figure 1. It is noted that in request frame $N$ denotes the number of sensor nodes.

Active period includes six kinds of frames (Table 1): beacon frame, request frame, emergency frame, statistical frame, data frame and acknowledge frame. We denote the payload size of each data frame as $L$. It is notable that the request frame has two subcategories: normal data request and emergency data request. The coordinator can recognize each kind of frame by the frame type byte. In the beacon-enable period, the star network is controlled by a coordinator, which regularly transmits a beacon packet for network synchronization. The star topology and short propagation distance in HBC platform make it possible to achieve more precise network synchronization with less energy consumed by high precision oscillators [40]. After synchronization, sensor nodes with data packet to send will start to prepare their respective request frames. The request frame is designed for sensor nodes to send their requests to the coordinator during predetermined time slots. Since the number of sensor nodes in a WBAN is always limited, it is applicable to give all the sensor nodes their default time slots by programming. The synchronization-time-division utilized in the protocol ensures that no collisions, idle listening or overhearing can occur in the request frame. The maximum number of request frames $N$ is initiated through programming and can be changed easily when needed. When a new sensor node is associated to the network, the coordinator will allocate a time slot for the new comer automatically so that it can send a request frame of its own in the allocated time slot. It is notable that the time slot allocated to the new sensor node is always after the old ones as shown in Figure 1.

**Table 1:** Format of the designed frames

Name of frame	Frame format					
Beacon frame	Frame type	Recipient address	Sender address	Time synchronization		CRC
	1 Byte	1 Byte	1 Byte	2 Bytes		2 Bytes
Request frame	Frame type	Recipient address	Sender address	Number of request slots		CRC
	1 Byte	1 Byte	1 Byte	1 Byte		2 Bytes
Statistical frame	Frame type	Recipient address	Sender address	Total time slots	Node serial number and time slots	CRC
	1 Byte	1 Byte	1 Byte	2 Bytes	2 $N$ bytes	2 Bytes
Data/emergency frame	Frame type	Recipient address	Sender address	Frame sequence	Data	CRC
	1 Byte	1 Byte	1 Byte	1 Byte	$L$ Bytes	2 Bytes
Acknowledge $L$ frame	Frame type	Recipient address	Sender address	Time synchronization	Node serial number and time slots	CRC
	1 Byte	1 Byte	1 Byte	2 Bytes	2 $N$ Bytes	2 Bytes

**Figure 2:**
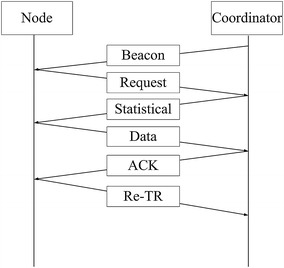
The workflow of the program. This figure demonstrates the working procedure of S-TDMA. To simplify the process, we take the situation when there is one sensor node and one coordinator as an example.

After receiving the request frames, the coordinator will add up all the requested time slots needed by the sensor nodes to form a statistical frame. Therefore, the statistical frame contains the total time slot request and different requests of all sensor nodes. Then, the coordinator sends the statistical frame with scheduling information to all sensor nodes, after which it prepares to receive data frames. All sensor nodes have the whole WBAN scheduling information and work accordingly. If no sensing event occurs after receiving the statistical frame, sensor nodes will set their radios into sleep mode until the next scheduled beacon frame comes. Otherwise, each sensor node will just wait until the granted time slot comes to send its data frame. Then, the coordinator will add up all the data frames from the sensor nodes, extract their information and compare it to that in the request frame, respectively. In this way, the coordinator determines which sensor nodes have experienced packet loss and arrange time slots for them in the acknowledge frame. The acknowledge frame will be sent to all the sensors nodes except those in the sleep mode. Each node will determine whether it has lost packet or not. If so, it will retransmit the lost data during the time slot allocated to it in the acknowledge frame (Re-TR period shown in Figure 1); if not, it will enter into sleep mode immediately. The duration of the data frame is adjusted by the coordinator based on the current traffic characteristics. To save energy, a period of inactivity is reserved for sensor nodes, allowing them to enter into sleep mode. Each data frame has a packet number assigned, so that the received packets are counted to maintain data integrity. Theoretically, S-TDMA protocol is compact and flexible. The workflow of the program is shown in Figure 2.

**Figure 3:**
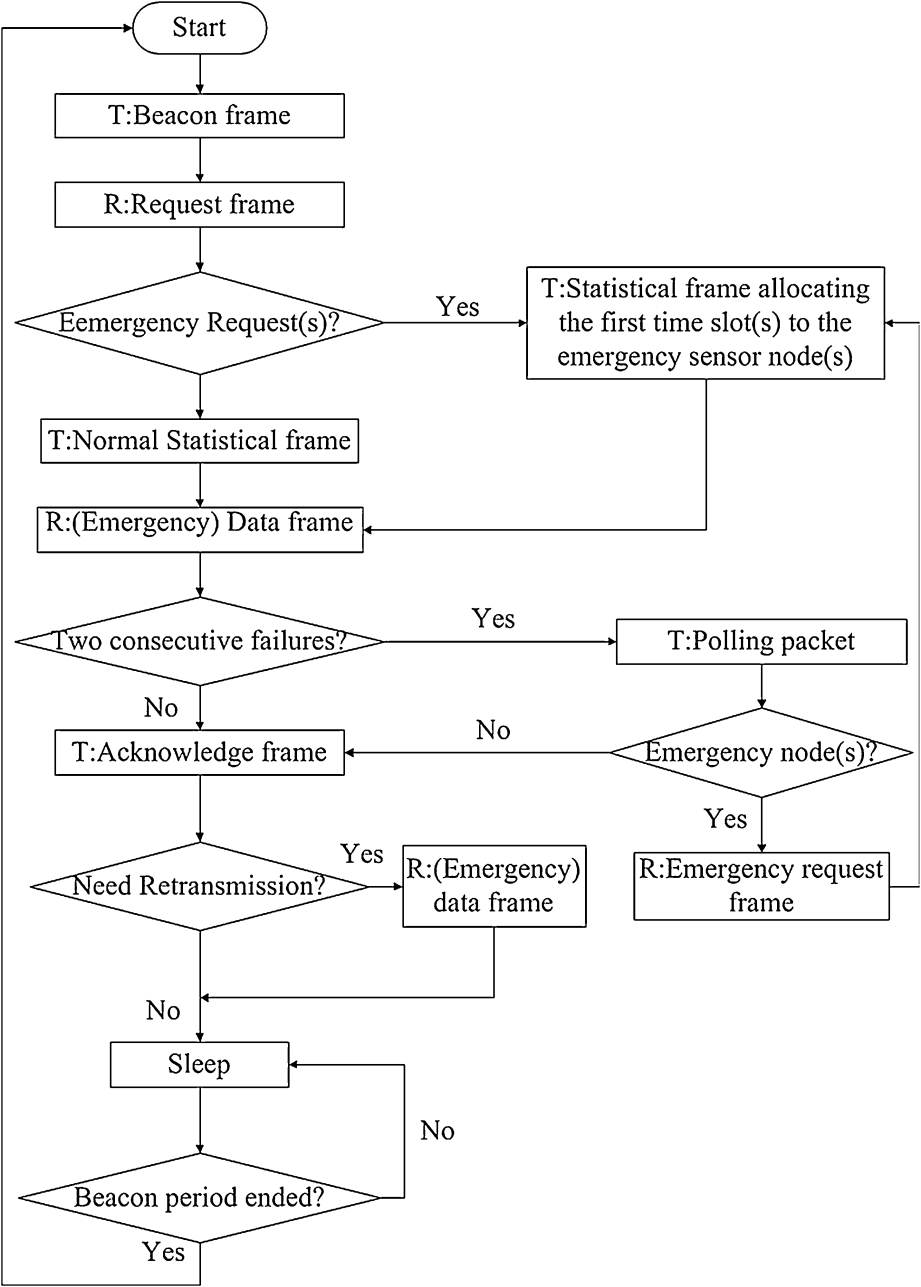
Flow chart of the emergency mechanism (T represents transmit, R represents receive). This figure demonstrates how S-TDMA handles burst traffic.

In addition, there is an emergency mechanism for vital signals to be transmitted. The emergency mechanism is shown in Figure 3. If a sensor node has an emergency frame to send, the coordinator will cut off the current processing and deal with the emergency node point to point immediately without waiting for the next beacon period. The coordinator can detect an emergency frame through the first byte of the frame. If the emergency data come before the sensor node send the request frame, the sensor node will send an emergency request frame to the coordinator. When allocating time slots in the statistical frame, the node with emergency will be given the first time slot. If there are more than one sensor nodes which have emergency data at this time period, the emergency nodes will be allocated the first few time slots. If the emergency data come when another sensor node is sending frames to the coordinator, the sensor node with emergency data will send the emergency request frame three times (the number of times can be set according to the working circumstances and channel characteristics). Inevitably, there will be two consecutive collisions (one time less that the set number of times) which can cause energy consumption, but it is well worth when delay is taken into consideration. When the coordinator detects two transmission failures, it will stop the current process and sent a polling packet to the sensor nodes. The polling packet will make sure whether there is an emergency node (or more than one emergency sensor nodes). Then, if there is an emergency node, the coordinator will arrange a slot to it during which the sensor node sent an emergency request again. When the coordinator receives the emergency request frame, it will react to the request immediately. When there are more than one emergency sensor nodes, the coordinator will arrange time slots according to the number of sensor nodes during which they send request to the coordinator. Then, the coordinator will react to the request. After the emergency, the coordinator will recover the processing according to the time period when the emergency comes. If the emergency occurs during the beacon frame, statistical frame or acknowledge frame period, the coordinator will resend a beacon frame, statistical frame or acknowledge frame to the sensor nodes; if the emergency occurs during the request frames, data frames or Re-TR period, the coordinator will ask the sensor nodes to retransmit the frames to it; if the emergency occurs during the sleeping period, it will wake up the coordinator to deal with it. In order to minimize the cost of the overhead, the frame format of request frame and statistical frame should be designed as simple as possible, so that all sensor nodes in WBAN can enter into sleep mode as soon as possible. The whole keynote and the design thinking are to reduce the service period of the radio transceiver and make the whole energy consumption to the least.

#### Dynamic time slot allocation

**Figure 4:**
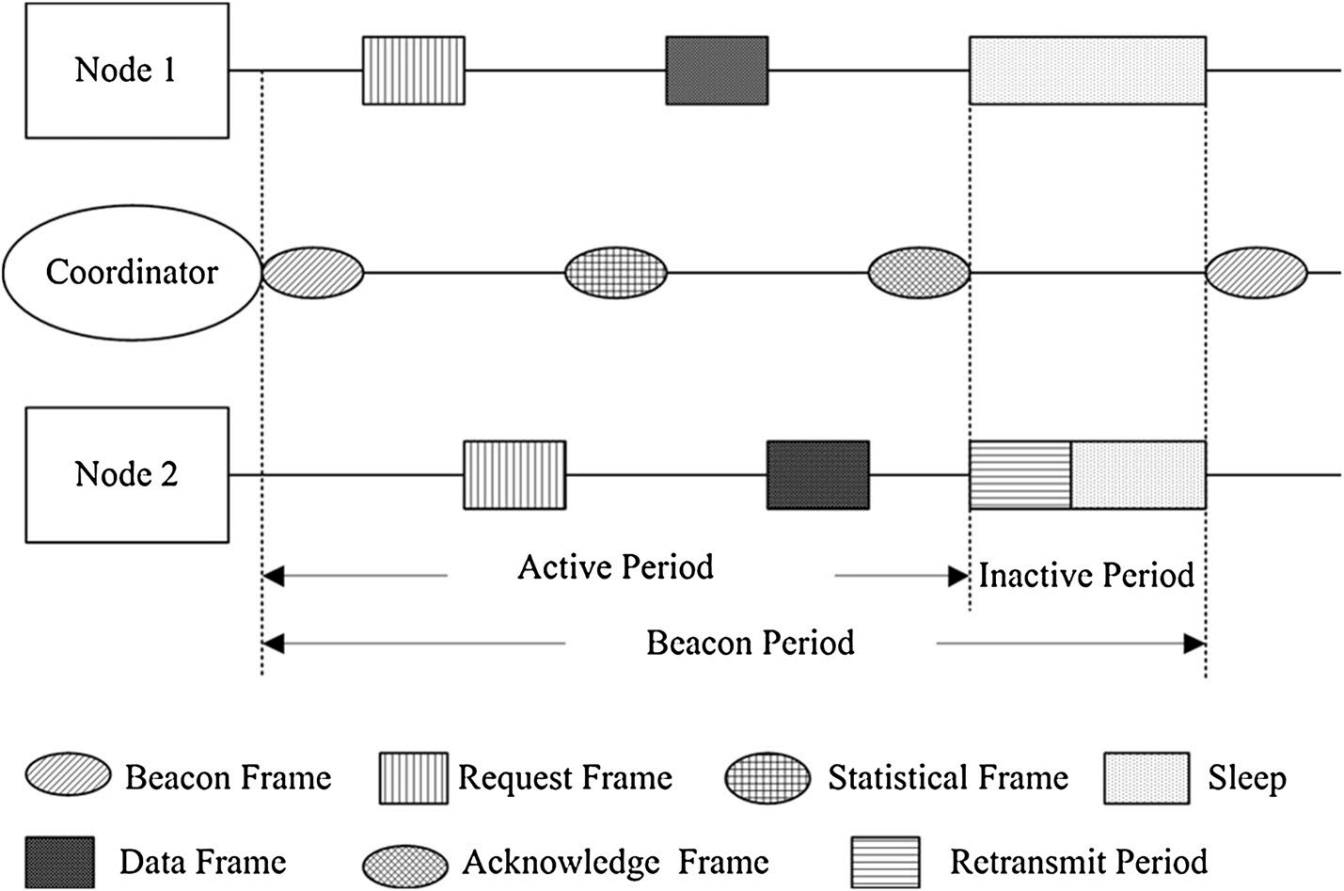
Dynamic time slot allocation scheme. This figure shows how the coordinator assigns time slot to each node in a situation where no emergency traffic occurs.

We use dynamic time slot allocation to handle changes in the load of the sensor nodes. Statistical frame allows the coordinator to allocate time slots in a flexible manner which compensates the cost of increased overhead resulting from using the request frame and statistical frame. The basic scheme is illustrated in Figure 4. Firstly, after powering on and initialization of the platform, the coordinator sends beacon frame to all sensor nodes (e.g. two nodes) for network synchronization. Secondly, each sensor node synchronize its real time clock (RTC) according to the information received in the beacon frame and then sends a request frame to the coordinator based on the number of data packets need to be sent. During the emergency slot, idle channel means that every nodes is in normal situation, while busy channel denotes emergency situation in the network. Thirdly, the coordinator processes all requests and sends scheduling information to all sensor nodes [41]. In this way, the structure of the superframe change dynamically according to the circumstances. Dynamic time slot allocation also reduces the active period in each beacon period, thus making WBAN more energy-efficient.

#### Analysis

We model the energy, delay and transmission efficiency performance for nodes having no-emergency traffic. We validate both the models with experimental results in the hardware platform. In this section, we derive analytical expressions for the energy consumption, delay and transmission efficiency. We assume that there is no emergency traffic and all the packets are transmitted successfully without any retransmission required.

*Energy analysis* We focus on the radio energy consumption of the coordinator and sensor nodes. There are four states: listen, transmit, receive and sleep for a radio device, and each state has different power consumption level. Accordingly, the total energy consumption, denoted as $E$, can be modelled by determining the fractional time it stays in each state per time unit. $P_{L}$, $P_{T}$, $P_{R}$ and $P_{S}$ are set as the power consumption in each state, and the time spent in each state for coordinator as $T_{ct}$ and $T_{cr}$ respectively, for sensor nodes as $T_{nt}$, $T_{nr}$, $T_{nl}$ and $T_{ns}$ respectively. We denote $T_{R}^{i}$ as the length of request frame for node $i$ and $T_{D}^{i}$ as the length of data frame.

The total energy consumption for the coordinator and sensor nodes:

1 $$ E = E_{c} + E_{nodes} $$

Energy consumption for the coordinator:

2 $$ E_{c} = P_{T} T_{ct} + P_{R} T_{cr} $$

Expected staying time during transmission for the coordinator:

3 $$ T_{ct} = T_{B} + T_{S} + T_{A} $$

Expected staying time during reception for the coordinator:

4 $$ T_{cr} = \sum\limits_{i = 1}^{N} {T_{R}^{i} } + \sum\limits_{i = 1}^{N} {T_{D}^{i} } $$

Energy consumption for sensor nodes:

5 $$ E_{nodes} = P_{T} T_{nt} + P_{R} T_{nr} + P_{L} T_{nl} + P_{S} T_{ns} $$

Expected staying time during listening for sensor nodes:

6 $$ T_{nl} = \sum\limits_{j = 1}^{N} {jT_{R}^{j} } - \sum\limits_{i = 1}^{N} {T_{R}^{i} } + \sum\limits_{j = 1}^{N} {\left( {N - j + 1} \right)T_{D}^{j} } - \sum\limits_{i = 1}^{N} {T_{D}^{i} } $$

Expected staying time during sleeping for sensor nodes:

7 $$ T_{ns} = T - T_{ct} - T_{cr} $$

The proposed protocol has five kinds of frames: beacon frame, request frame, statistical frame, data frame and acknowledge frame. We denote $T$ as superframe interval, and the length of each frame as $T_{B}$, $T_{S}$ and $T_{A}$ respectively. Equation (1) presents the total energy consumption for the coordinator and sensor nodes. Equation (2) models energy consumption for the coordinator, which consists of transmitting and receiving energy. Equation (3) presents expected staying time during transmission for the coordinator, which transmits a beacon frame, a statistical frame and an acknowledge frame. The coordinator receives request frames and data frames transmitted by sensor nodes, hence, expected staying time during reception for the coordinator as shown in Eq. (4). Energy consumption of sensor nodes is shown in Eq. (5), which consists of transmitting, receiving, listening and sleeping energy. Expected staying time during transmission for sensor nodes equals the expected staying time during reception for the coordinator. Expected staying time during reception for sensor nodes of expected staying time during transmission for the coordinator is $N$ times. In Eq. (6), the expected listen time for sensor nodes includes the listen time when sensor nodes transmit request frames and data frames to the coordinator. The listen time is concerned with the number of sensor nodes and data frames. Equation (7) presents expected staying time during sleeping for sensor nodes.

*Average latency analysis* Here, we model the expected delay period within a superframe period.

The data latency for node $i$:

8 $$ D_{i} = T_{B} + \sum\limits_{i}^{N} {T_{R}^{i} } + T_{S} + \sum\limits_{i = 1}^{i} {T_{D}^{i} } $$

The data latency for total nodes:

9 $$ D_{total} = T_{B} + \sum\limits_{j = 1}^{N} {jT_{R}^{j} } + T_{S} + \sum\limits_{j = 1}^{N} {\left( {N - j + 1} \right)T_{D}^{j} } $$

Active period includes six kinds of frames: beacon frame, request frame, emergency frame, statistical frame, data frame and acknowledge frame. We have assumed there is no emergency traffic. Hence, we need consider the latency of beacon frame, request frame, statistical frame and data frame. Equation (8) presents the transmission latency for node $i$. We denote $T_{R}^{i}$ as the length of request frame for node $i$ and $T_{D}^{i}$ as the length of data frame. Equation (9) models the transmission latency for total nodes. In a superframe period, the coordinator transmits a beacon packet for network synchronization, and sends the statistical frame with scheduling information to all sensor nodes. Accordingly, the transmission latency for total nodes includes a beacon frame period and a statistical frame period.

*Transmission efficiency analysis* There is no special definition for transmission efficiency. We use a definition of the transmission efficiency as the ratio between the number of useful data bytes and the total number of bytes transmitted in a certain period.

The number of useful data bytes transmitted by the sensor nodes:

10 $$ M_{un} = \sum\limits_{i = 1}^{N} {H_{i} L_{x} T} $$

We obtain the following formulate for the transmission efficiency:

11 $$ \eta = \frac{{M_{useful} }}{{M_{total} }} $$

We set that the node $i$ transmits $H_{i}$ data frames, and denote the payload size of each data frame as $L_{x}$. The useful data is collected by the coordinator and sensor nodes, and the useful data received by the coordinator equals the useful data transmitted by the sensor nodes. The total number of bytes transmitted includes the number of beacon frame bytes, request frame bytes, statistical frame bytes, acknowledge frames bytes and the bytes aside from the data packet in a data frame.

### Protocol implementation

#### Experimental platform

**Figure 5:**
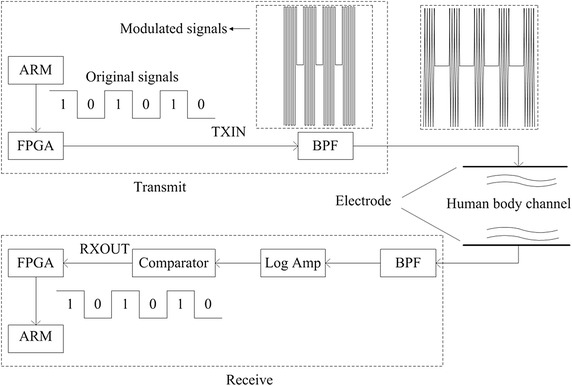
Diagram of the HBC transceiver architecture. This figure illustrates how the data transmit from the coordinator (the *dashed box* named “transmit”) to each sensor (the *dashed box* named “receive”).

The functional block diagram of HBC experimental platform is illustrated in Figure 5. It includes battery-powered transmitter (TX) and receiver (RX) modules. Each TX/RX module is provided with a signal electrode. The signal electrode is coupled with the human body by capacitive coupling instead of direct electrical contact. An electrode is made up of a piece of nickel conductive cloth encapsulated into electrical tape and connected to the module through a short RF cable. The transceiver uses OOK for modulation and demodulation through FPGA in a half-duplex manner. In addition, the transmission frequency is 40 MHz with transmission level of 3 V.

**Figure 6:**
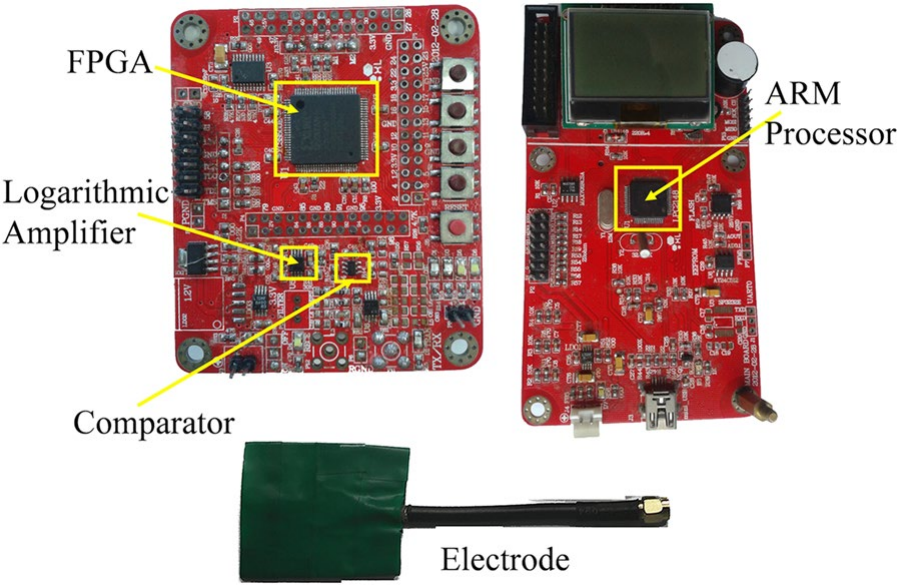
Figure for HBC development platform. This figure aims to demonstrate the printed circuit board (PCB) of our independently developed HBC platform.

As shown in Figure 5, the ARM processor is in charge of system controlling, data gathering and processing. The six kinds of frames (shown in Table 1) are formed in ARM, not including cyclic redundancy check (CRC) bytes which are formed in FPGA. The MAC protocol tasks and the default time slots are also implemented in ARM on the sensor nodes through programming. Embedded C programming language is used in programming which is debugged in the compiler environment Keil uvision4. The program is downloaded to ARM through J-link. The workflow of the program is shown in Figure 2. For data transmission, the ARM processor reads data from the secure digital memory card (SD card) and then modulate it into a 40 MHz square wave which falls within the specified frequency band range for HBC [42]; the square wave is sent to FPGA through serial peripheral interface (SPI), after which it is filtered by a band pass filter (BPF) and becomes a sine wave of the same frequency. The sine wave is passed to the signal electrode and transmit through our body. Our body is able to transmit the signals through capacitive coupling. As to the RX part, the signal is gathered by the input electrode and then filtered by a band-pass filter (BPF); then signal is demodulated by a logarithmic amplifier; at last, the signal is sent to the comparator through which the original signal is reverted; Clock and data recovery (CDR) is done in FPGA. The figure for experimental platform is shown in Figure 6. The coordinator as well as each HBC slave node is equipped with an electrode, data rates supported by this platform are up to 10 Mbps, meeting the demands of the latest entertainment and healthcare services.

#### Experimental scenarios and parameters setting

**Figure 7:**
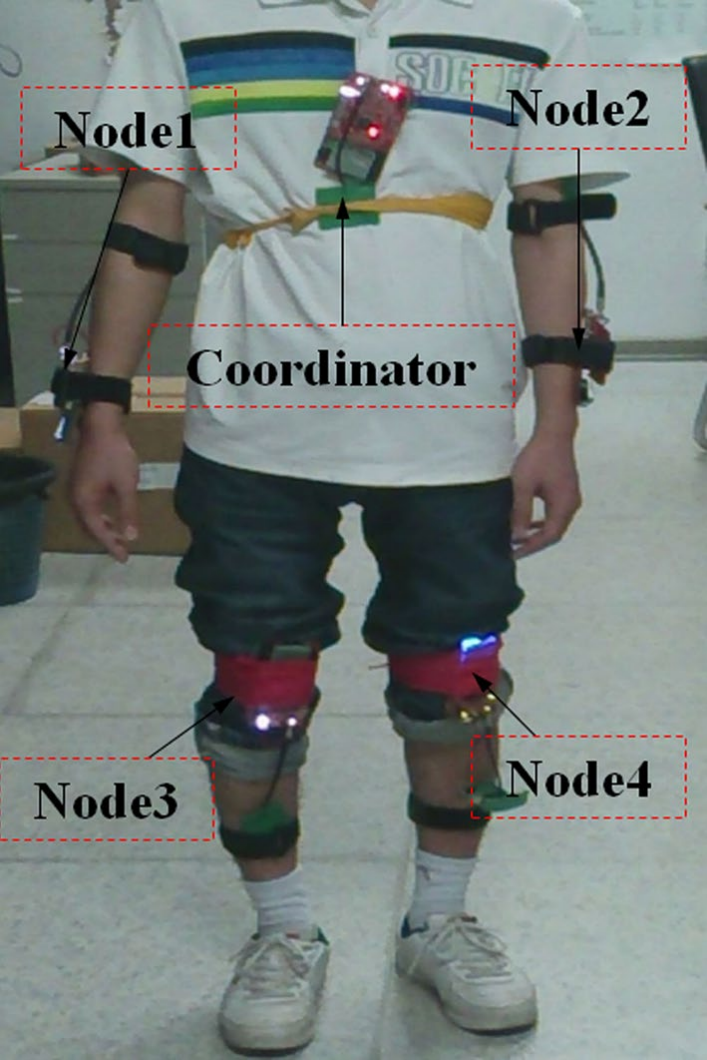
HBC experimental scenarios. A coordinator and four sensor nodes are fastened on the body of the volunteer. To collect the information of moving, sensor nodes are fastened on the arms and legs of the volunteer.

To evaluate the performance of our MAC protocol in burst traffic scenarios, a WBAN-based HBC network is built using the presented development platform. We compare it with other famous MAC protocols, such as PB-TDMA protocol, LMAC protocol and IEEE 802.15.6 CSMA/CA protocol, all of which are integrated into our platform in turn. The aim of the experiment is to analyze human movement [43]. In this application the sampling rate is 80–100 Hz when the subject is walking (1.5 ± 0.28 m/s) [43]; 120–150 Hz when the subject is running (1.9 ± 0.39 m/s) [43]; and 200–250 Hz for fall event. These three types of sampling rate create different numbers of data packets to be sent in the beacon period. In the experiment, we utilized one coordinator and four nodes to test S-TDMA protocol, as shown in Figure 7. The experiment had a total duration of 20 s: in the first 10 s the subject was walking; in the next 9 s the subject was running; finally, a fall event was performed at the 20th second. We build in an inertial measurement unit (IMU) in our sensor nodes to collect motion data of the object (in our case a person).

We set the MAC parameters values for these four protocols according to IEEE standard for local and metropolitan area networks—part 15.6: wireless body area networks (WBAN) [44], several important parameters are listed in Table 2.

**Table 2:** MAC parameters values for these four protocols

Parameter	Value
mBAckLimit	8
mCSMATxLimit (CSMA/CA only)	2 for UP ≤5 or 4 for UP ≥6
mG-AckDataSubtype	1,111 (binary)
mMaxFragmentCount	8
mMaxBANSize	64
mScheduledAllocationAborted	32
mTimeOut	30 μs
mUnscheduledAllocationAborted (CSMA/CA only)	32
mUnscheduledNoResponseLimit (CSMA/CA only)	3
pAllocationSlotMin	500 μs
pMaxFrameBodyLength	255 octets

It is notable that all the parameters in Table 2 are adopted in these four protocols except for those parameters with CSMA/CA only which are only used in IEEE 802.15.6 CSMA/CA protocol. The meaning and usage of those parameters are detailed in [44].

## Results and discussion

### Energy consumption comparison

We set the size of each data packet to 12 bytes. According to the parameters settings mentioned above, a sensor node will receive 960–1,200 bytes per second when the subject is walking, 1,440–1,800 bytes per second when running, 2,400–3,000 bytes per second when falling down. Considering that the payload size of each data frame is 122 bytes, a sensor node need to send 10, 15, 25 data frame per second, respectively. The size of the beacon frame, request frame, statistical frame, data frame and acknowledge frame are 7, 6, 15, 128, and 15 bytes, respectively.

**Figure 8:**
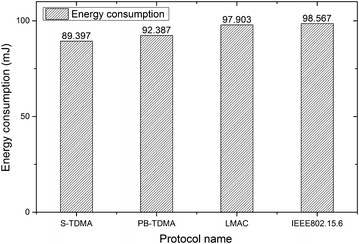
Comparison of energy consumptions among the four protocols implemented on our development platform. This figures shows the energy consumption of the coordinator and four sensor nodes in 20 s. Since there are 15 experiments, average value of these experimental results are given.

We set the nodes to transmit data for 5 min, and measured the total consumed energy by a power meter, thus achieving the value of consuming energy (both the transmitter and the four receivers) per bit. It is notable that the “5 min” is divided into fifteen “20 s” according to the “total duration” aforementioned. Then, we calculated the total energy consumption in 20 s with measured results. We measured the energy consumption in other scenes in the same way. Results are shown in Figure 8.

It is clear that, S-TDMA demonstrates lower energy consumption compared to the other three protocols. PB-TDMA uses a preamble for data slot allocation. Therefore, when many nodes activate their destination nodes at the same time, preamble collisions will occur. LMAC is more energy-consuming than S-TDMA for the reason that its overhead is larger than that of S-TDMA. IEEE 802.15.6 employ a slotted CSMA/CA scheme where nodes compete for transmission opportunity in the contention access period (CAP) while our proposed protocol utilized special time slots to collect requests from sensor nodes.

It is notable that those experimental results shown above were obtained from the average value of 15 experiments (each experiment lasts for 20 s).

In addition, dynamic changes in the number of the nodes associated to a given WBAN will lead to corresponding dynamic change in the total number of slot requests. In a star-topology WBAN driven by one coordinator, if there is no scheduling applied, the coordinator would waste energy in waiting to receive data during association or de-association of a node.

**Figure 9:**
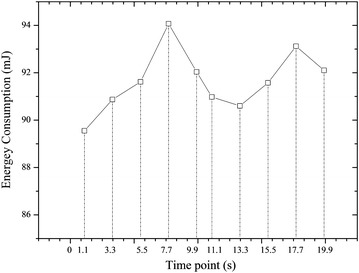
Measured results of energy consumption with emergency events happens at ten different time points. This figures shows the energy consumption of the coordinator and four sensor nodes when there is an emergency.

To evaluate the emergency mechanism of S-TDMA, we choose Node 3 in Figure 7 as the emergency one, in which we put a data packet (18 bytes) in its SD card. Through programming, we set the sensor node to send this “Emergency data packet” at certain time point. Since emergency event can happen during any time in the beacon period, we have performed ten times of experiments (each experiment lasts for 20 s as mentioned above) with different time points at which the emergency event take place. Since one beacon period lasts for one second, we select five time points with equal time interval 0.1, 0.3, 0.5, 0.7 and 0.9 s to demonstrate the randomness of emergency event. In addition, an experiment period includes 20 beacon periods, so we select 10 time points, i.e. 1.1, 3,3, 5.5, 7.7, 9.9, 11.1, 13.3, 15.5, 17.7, and 19.9 s, respectively to simulate the three different movement stages (walking, running, falling). Measured results are shown in Figure 9.

We use the measured data in Figure 8 to calculate energy consumption when the number of sensor nodes increases. Take S-TDMA as an example, we measured that when the number of sensor node is four, the energy consumption is 89.397 mJ. We calculate that the number of bits transmitted during one experimental period (20 s) is 2,167,200, which we will explain in the next paragraph. Let 89.397 mJ divided by 2,167,200, we will achieve that in our platform, the total energy needed to transfer one bit of information (i.e. the sum of energies drawn both by transmitter and receiver) is about 41.25 nJ.

**Figure 10:**
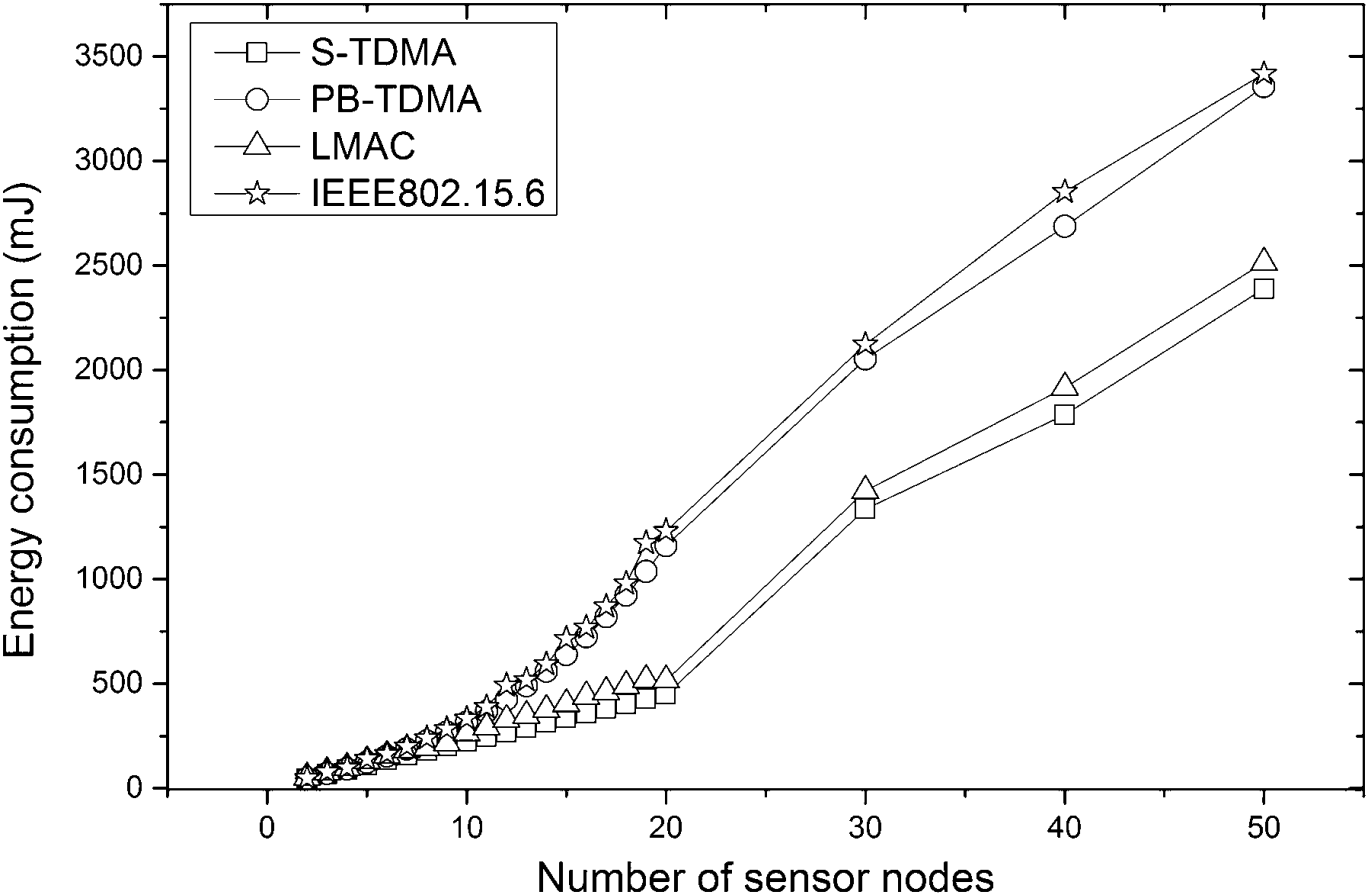
Comparison of energy consumptions among the four protocols with the number of nodes increased. This figure illustrates that the advantage of S-TDMA in saving energy becomes greater as the number of sensor nodes increase.

The method to calculate the energy consumption values for increasing numbers of sensor nodes is illustrated as follows. First, the number of beacon frames, request frames, statistical frames, data frames and acknowledge frames transmitted in an experimental period is calculated. Second, according to the size of each frame mentioned above, we get the number of bits transmitted under ideal circumstances when all the packets are transmitted successfully in one time (i.e. no retransmission needed). Then, we set the bit error rate (BER) to $2. 5\times 1 0^{-3}$ (the number is set according to network density and channel characteristics [45]). In this way, we get the approximate number of bits transmitted in an experimental period. At last, let the number of bits multiply by 41.25 nJ, we achieve the energy consumption values for increasing number of sensor nodes (shown in Figure 10).

It is obvious that the energy consumption of all protocols increases with sensor nodes while S-TDMA remains the lowest among the four protocols. The more sensor nodes associated, the more obvious the advantage of S-TDMA is. It is necessary to mention that all the sensor nodes are attached to the same coordinator. With respect to the MAC parameter listed in Table 2, the maximum sensor nodes in a WBAN is 64. To demonstrate the advantages in energy efficiency and data latency as the number of sensor nodes increases, we set the largest number of sensor nodes in our calculation to 50.

### Average latency

**Figure 11:**
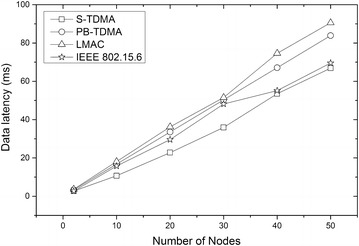
Comparison of the data latency among four protocols. This figure shows that the flexible time slot allocation mechanism makes S-TDMA the most competitive protocol when handling burst data traffic.

We calculate the data latency of S-TDMA and the other three protocols according to the average latency analysis. Figure 11 shows a comparison of data latency among four of the protocols. Therefore, the application of flexible time slot allocation in the proposed protocol with the request frame and statistical frame allow it to achieve low data latency with on-demand traffic.

### Transmission efficiency

**Figure 12:**
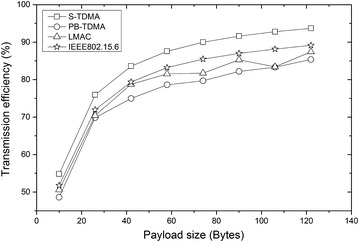
Comparison of transmission efficiency among the four protocols with the payload size of data frame increased. This figure shows that by using request frame and statistical frame, S-TDMA can get higher transmission efficiency in comparison with the other protocols.

Sending, receiving, and listening for overhead can be a source of energy wastage [40]. To measure the influence of overhead of the superframe, we calculated the transmission efficiency of S-TDMA and that of the other three protocols. Transmission efficiency counts not only with the data bits but also with the overhead that makes use of the channel. Control packet overhead is a major source of energy that we consider here. Sending, receiving, and listening for control packets consume energy. Since, control packets do not directly convey useful application data; they also reduce the effective throughput. In this experiment, the transmission overhead consists of beacon frame, request frame, statistical frame, acknowledge frame and the bytes aside from the data packet in a data frame. The following assumptions are considered for calculation: (a) the channel is noise free; (b) there is no data loss during the transmission, namely: no retransmission is needed. We calculate the transmission efficiency of S-TDMA and the other three protocols according to the transmission efficiency analysis. The transmission efficiency of all the four protocols is illustrated in Figure 12. Though the utilization of request frame and statistical frame can inevitably lead to an increase in overhead, the transmission efficiency of S-TDMA is still higher than the other three protocols.

### Comparison

Table 3 shows the comparison of energy consumption, data latency and transmission efficiency among the four protocols when the number of sensor nodes is four and the payload size is 122 bytes (as mentioned in the parameters settings). Based on all the experimental results, we can draw three conclusions as follows. First, with respect to power efficiency in on-demand traffic network, S-TDMA in the paper outperformed the other three protocols; second, S-TDMA has lower data latency and higher transmission efficiency; third, the incense in the number of sensor nodes can highlight the advantage of S-TDMA.

**Table 3:** Comparison of energy consumption, data latency and transmission efficiency among the four protocols

	Energy consumption (mJ)	Data latency (ms)	Transmission efficiency (%)
S-TDMA	89.397	6.27	93.67
PB-TDMA	92.387	6.71	85.37
LMAC	97.903	7.92	87.41
IEEE 802.15.6	98.567	7.67	89.15

### Future work

The proposed S-TDMA protocol demonstrates good performance in the scenario where burst traffic is often triggered rapidly with low power consumption and low delay. However, the versatility of S-TMDA should be evaluated in a range of WBAN application environments in future work, and the improvements of MAC protocols for other scenarios should also be considered.

## Conclusions

In this paper, an S-TDMA protocol with multi-constrained (energy, delay, transmission efficiency and emergency management) is customized for HBC. A novel superframe structure is proposed that allows the normal traffic to be transmitted without competition. Meanwhile, a dynamic time slot allocation mechanism is presented to satisfy the emergency traffic.

The performance of S-TDMA was compared with that of PB-TDMA, LMAC and IEEE 802.15.6. The results demonstrate that the S-TDMA behaves better, which successfully meets the delay and transmission efficiency requirements of HBC while keeping a low energy consumption.

## Abbreviations

WBAN: wireless body area networks
PHY: physical
HBC: human body communication
MAC: media access control
TDMA: time division multi access
WSNs: wireless sensor networks
CSMA: carrier sense multiple access
CSMA/CA: carrier sense multiple access/collision avoidance
CCA: clear channel assessment
PB-TDMA: preamble-based TDMA
LMAC: lightweight MAC
